# Circular RNA profiling distinguishes medulloblastoma groups and shows aberrant *RMST* overexpression in WNT medulloblastoma

**DOI:** 10.1007/s00401-021-02306-2

**Published:** 2021-04-17

**Authors:** Daniel Rickert, Jasmin Bartl, Daniel Picard, Flavia Bernardi, Nan Qin, Marta Lovino, Stéphanie Puget, Frauke-Dorothee Meyer, Idriss Mahoungou Koumba, Thomas Beez, Pascale Varlet, Christelle Dufour, Ute Fischer, Arndt Borkhardt, Guido Reifenberger, Olivier Ayrault, Marc Remke

**Affiliations:** 1 Division of Pediatric Neuro-Oncogenomics, German Cancer Research Center (DKFZ), Heidelberg, Germany; 2 German Cancer Consortium (DKTK), Partner Site Essen/Düsseldorf, Düsseldorf, Germany; 3 Department of Pediatric Oncology, Hematology, and Clinical Immunology, University Hospital Düsseldorf and Medical Faculty, Heinrich Heine University, Düsseldorf, Germany; 4 Institute of Neuropathology, Düsseldorf and Medical Faculty, University Hospital, Heinrich Heine University, Düsseldorf, Germany; 5 Institut Curie, CNRS UMR, INSERM, PSL Research University, Orsay, France; 6 CNRS UMR 3347, INSERM U1021, Université Paris Sud, Université Paris-Saclay, Orsay, France; 7 Department of Control and Computer Engineering, DAUIN, Politecnico di Torino, Torino, Italy; 8 Department of Pediatric Neurosurgery, Necker Hospital, APHP, Université Paris Descartes, Paris, France; 9 Medical Faculty, Department of Neurosurgery, Heinrich Heine University, Düsseldorf, Germany; 10 Department of Neuropathology, GHU Paris-Neurosciences, Sainte-Anne Hospital, 75014 Paris, France; 11 Department of Pediatric and Adolescent Oncology, Gustave Roussy, Villejuif, France; 12 INSERM, Molecular Predictors and New Targets in Oncology, University Paris-Saclay, Villejuif, France

Medulloblastoma is the most common malignant brain tumor in childhood [7]. Molecular classification into WNT, SHH, Group 3, and Group 4 of medulloblastoma refines surveillance of tumor predisposition syndromes, improves risk stratification to (de-)escalate therapeutic interventions, and sets the stage for targeted therapies [7]. Discrimination between Group 3 and Group 4 medulloblastoma remains challenging with partly inconsistent group annotation using DNA methylation data [1], gene expression profiling [8] or (phospho-)proteomics [3]. Thus, studies are increasingly integrating two or more molecular layers for accurate group assessment of this heterogeneous disease [1, 8].

Circular RNAs (circRNA) recently emerged as promising biomarkers with cell type- and developmental stage-specific expression patterns in human tissues and cancers [10]. They are not easily degraded by exonuclease RNase R, are long-lived and are widely detected in body fluids [10]. Based on these unique properties, circRNAs may serve as clinically useful diagnostic biomarkers. We developed a bioinformatic approach called “circs”, combining three circRNA detection pipelines (find_circ [5], DCC [2], and CIRCexplorer [11]) reducing the false-positive rate compared to single in silico circRNA detection method.

We could detect circRNAs with higher expression in medulloblastoma (Figures S1a and S1b, Table S1), but most circRNAs were significantly lower expressed compared to fetal brain tissue samples as previously reported (Fig. 1a) [10]. In contrast to the previous indications that non-coding RNA expression profiles were not sufficient to distinguish groups [9], unsupervised hierarchical clustering revealed the four core groups using the top 500 most differentially expressed circRNAs in our discovery cohort (*n* = 38, Fig. 1b, Table S2), and in our non-overlapping validation cohort (*n* = 35; Fig. 1c, Table S3). We confirmed medulloblastoma classification of the discovery cohort based on the previous similarity network fusion (SNF) analysis (Fig. 1d, data obtained from methylome, transcriptome, and proteome [3]) and using circRNA-based grouping in our discovery cohort (Fig. 1e) and in our validation cohort (Fig. 1f).

**Fig. 1:**
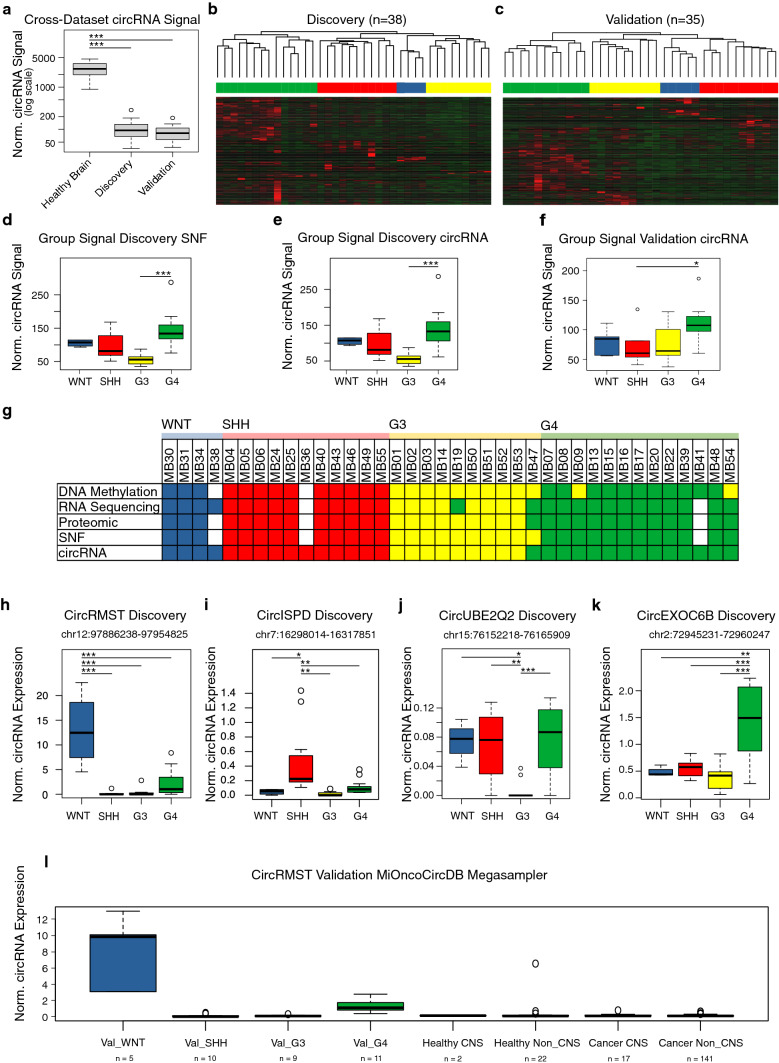
Circular RNA expression profiles define medulloblastoma groups. **a** Circular RNA (circRNA) signal across fetal brain controls (healthy, *n* = 12), discovery (*n* = 38) and validation medulloblastoma (MB) cohort (*n* = 35). **b**, **c** Heatmap of top 500 differentially expressed circRNAs in discovery (**b**) and in validation data set (**c**), hierarchical clustering by average Pearson dissimilarity, circRNA MB groups: *WNT* blue, *SHH* red, *G3* yellow, *G4* green. **d** Circular RNA signals across discovery data set similarity network fusion (SNF) MB groups. **e** Circular RNA signal across discovery data set (circRNA MB groups). **f** Circular RNA signal across validation data set (circRNA MB groups). **g** Medulloblastoma grouping according to [3] and circRNA data in discovery data set. MB36 was diagnosed as SHH-MB in the routine diagnostic setting. White square = data not available. **h–k**. Boxplot of circRMST, circISPD, circUBE2Q2, and circEXOC6B in discovery data set with circRNA-based groups and genomic coordinates. **l** Megasampler with normalized circRMST expression in validation data set with the circRNA groups (Val_WNT = WNT validation data set; Val_SHH = SHH validation data set; Val_G3 = G3 validation data set; Val_G4 = G4 validation data set) and MiOncoCircDB (four categories: healthy_CNS, healthy_non_CNS, cancer_CNS, cancer_non_CNS, details see Tables S9 and S10). Tukey’s HSD adjusted *p* values: *p < 0.05; **p < 0.01; ***p < 0.001

Using only circRNA expression profiles of the top 500 most differentially expressed circRNAs in our discovery cohort, we were able to reliably separate the four core groups almost as accurately as using SNF integrating multi-omics data (34/35 = 97.14% concordance; adjusted rand index = 0.91, *p* = 0.001; Fig. 1g).

To discern group-specific and subtype-specific circRNA biomarkers, we performed differential expression analysis of the circRNA-defined groups (Fig. 1h–k, Figures S2a–e, S3a–d) and of DNA methylation-defined subtypes (Figure S4a–d, Table S4). Significant and consistent up- or downregulations of circRNAs were identified in both cohorts for WNT (*n* = 81; Table S5, Figs. 1h, S2a, S3a), SHH (*n* = 7; Table S6, Figs. 1i, S2b, S3b), and Group 4 (*n* = 13; Table S7, Figs. 1k, S2d, S3d), while Group 3-specific circRNAs were not consistently detected (Table S8, Figs. 1j, S2c, S3c). Notably, a circRNA derived from the *rhabdomyosarcoma 2-associated transcript* (*RMST*) locus (chr12: 97886238–97954825) was aberrantly overexpressed in WNT medulloblastomas compared to the other groups (*p* < 0.001; Figs. 1h, S2a). *RMST* is a well-known long non-coding RNA, which is exclusively expressed in brain tissue [6], and mostly present in circular isoforms [4]. Using a published cohort MiOncoCircDB consisting of circRNAs across over 2000 cancer samples [10], we validated circRMST as a highly reproducible and specific biomarker for WNT medulloblastoma (Fig. 1I).

In conclusion, we demonstrate a powerful and reliable method for molecular classification using our pipeline *circs* without the necessity to include additional information layers. Thus, circRNA-based biomarkers may help to improve diagnostic and therapeutic approaches in this highly aggressive disease.

## Supplementary Information

Below is the link to the electronic supplementary material.

Supplementary file1 (DOCX 21 KB)

Supplementary file2 (PDF 33 KB)

Supplementary file3 (PDF 2997 KB)

Supplementary file4 (PDF 23 KB)

Supplementary file5 (PDF 618 KB)

Supplementary file6 (XLSX 306 KB)

Supplementary file7 (XLSX 1713 KB)

Supplementary file8 (XLSX 1493 KB)

Supplementary file9 (XLSX 3395 KB)

Supplementary file10 (XLSX 26 KB)

Supplementary file11 (XLSX 10 KB)

Supplementary file12 (XLSX 12 KB)

Supplementary file13 (XLSX 14 KB)

Supplementary file14 (XLSX 15 KB)

Supplementary file15 (XLSX 10 KB)
