# Supplementary material for: Circular RNA profiling distinguishes medulloblastoma groups and shows aberrant RMST overexpression in WNT medulloblastoma
Source: Acta Neuropathol. 2021 Apr 17;141(6):975–8. doi: 10.1007/s00401-021-02306-2 (PMC8113310; doi:10.1007/s00401-021-02306-2)
Supplement: Supplementary file 1 — Supplementary file1 (DOCX 21 KB) [file 401_2021_2306_MOESM1_ESM.docx]

**Materials and Methods**

*Patient samples and RNA sequencing pre-processing*

All medulloblastoma samples were collected following written informed consent. Approval for the study was obtained by the internal review board at the Necker Hospital for Sick Children (Paris, France, IRB approved protocol number DC-2009-955 for tumor banking) and by the internal review board of the Medical Faculty at Heinrich Heine University Düsseldorf (study numbers 3005 and 2018-45-FmB). All samples analyzed in this study were collected from newly diagnosed medulloblastoma. For the discovery (Table S2) (EGA: EGAD00001004327) and the validation cohort (Table S3), total RNA was prepared and sequenced as previously described [3]. Raw RNA sequencing data were demultiplexed based on unique adapter sequences and converted to fastq format using the bcl2fastq package

(<https://support.illumina.com/sequencing/sequencing_software/bcl2fastq-conversion-software.html>),

ensuring adapter sequences were not masked. Sequencing read quality was assessed using the FastQC sequencing quality control package (https://www.bioinformatics.babraham.ac.uk/projects/fastqc) and MultiQC (https://github.com/ewels/MultiQC). All samples passed all quality controls.

*Circular RNA detection using circs workflow*

Circular RNAs were quantified with a three pipelines workflow based on the reference genome hg19 including DCC [1], find_circ [4] and CIRCexplorer1 [6]. We named this approach *circs*. Briefly, *circs* (<https://gitlab.com/daaaaande/circs>) uses automated versions of the DCC, find_circ and CIRCexplorer1 (the automated version includes a non-default choice of STAR [2] as the aligner) pipelines, overlaps the data to minimize false positives and normalizes the final data output. First, each circRNA detection pipeline was run according to the input data format (here both datasets were paired end). Second, each of the three circRNA output data were filtered to include only circRNAs that are detected with at least two junction reads in at least one sample. Third, voting includes the overlap of the three filtered output tables: only circRNAs that were present in all three filtered output tables are accepted for further analysis. In a last step, the DCC quantifications of accepted circRNAs were normalized to DCC circRNA backsplice junction reads per million total RNA sequencing reads to ensure comparability across data sets. The normalized, filtered and voted DCC circRNA quantifications were used for downstream analysis.

*Data processing*

All downstream analyses were performed in R [5]. For clustering, the data were first normalized, filtered for the top 500 diverging (highest standard deviation across all samples) circRNAs and then clustered according to Pearson dissimilarity (1- (average Pearson correlation) [R package heatmap.2]. For statistical comparison of circRNA expression across medulloblastoma groups and subtypes, ANOVA was used with Tukey’s HSD as post-hoc test. All p-values shown in the manuscript are adjusted p-values.

*MiOncoCircDB comparisons*

To compare *circs* data to MiOncoCircDB expression data, *circs* validation data CIRCexplorer1 output was normalized to the median number of RNA sequencing reads in the dataset to ensure comparability between the two datasets (validation cohort: 43 million RNA sequencing on-target reads on average). MiOncoCircDB circRNA data set was downloaded from the MiOncoCircDB webpage (<https://mioncocirc.github.io/download/>) and normalized the same manner like before.

**References of methods**

1. Cheng J, Metge F, Dieterich C (2016) Specific identification and quantification of circular RNAs from sequencing data. Bioinformatics 32:1094–1096. https://doi.org/10.1093/bioinformatics/btv656
2. Dobin A, Davis CA, Schlesinger F et al. (2013) STAR: ultrafast universal RNA-seq aligner. Bioinformatics 29:15–21. https://doi.org/10.1093/bioinformatics/bts635
3. Forget A, Martignetti L, Puget Set al. (2018) Aberrant ERBB4-SRC Signaling as a Hallmark of Group 4 Medulloblastoma Revealed by Integrative Phosphoproteomic Profiling. Cancer Cell 34:379-395.e7. https://doi.org/10.1016/j.ccell.2018.08.002
4. Memczak S, Jens M, Elefsinioti Aet al. (2013) Circular RNAs are a large class of animal RNAs with regulatory potency. Nature 495:333–338. https://doi.org/10.1038/nature11928
5. The R Core Team (2017) R: A Language and Environment for Statistical Computing. https://www.R-project.org/
6. Zhang X-O, Wang H-B, Zhang Y et al. (2014) Complementary Sequence-Mediated Exon Circularization. Cell 159:134–147. https://doi.org/10.1016/j.cell.2014.09.001

**Figure Legend Supplementary Figures**

**Supplementary Figure S1: Circular RNA expression in medulloblastoma**

**a+b**. Boxplots of normalized circRNA expression in combined medulloblastoma (MB) cohorts (discovery and validation data sets together) *versus* healthy normal brain samples. CircFIRRE (b; Student`s t-test p=3.24x10^-5^) and circRNF220 (a; Student`s t-test p=1.16x10^-7^).

**Supplementary Figure S2: Circular RNA biomarkers in the different medulloblastoma groups of validation cohort**

**a-d**. Boxplots of normalized circRNA expression respectively in validation data set with methylome- and transcriptome-based groups in medulloblastoma (MB). CircRMST (a), circISPD (b), circUBE2Q2 (c) and circEXOC6B (d). Tukey`s HSD adjusted p-values: *=p<0.05; **=p<0.01; ***=p<0.001 (details see Tables S5, S6, S7 and S8). **e.** Corrplot of merged validation and discovery cohort of circRNA data. Pie charts and filling colors represent Pearson correlation of all circRNAs. Sample order according to circRNA-based MB groups.

**Supplementary Figure S3: Circular RNA biomarkers in the different medulloblastoma groups of combined cohorts**

1. **-h**. Boxplots of normalized circRNA expression in combined medulloblastoma (MB) cohorts (discovery and validation data sets together) *versus* healthy normal brain samples. circPATJ (a), circEYA1 (b), circEYS (c) and circRPH3A (d). Tukey`s HSD adjusted p-values: *=p<0.05; **=p<0.01; ***=p<0.001.

**Supplementary Figure S4: Circular RNA biomarkers in the different medulloblastoma subtypes**

**a+b**. Heatmap of top 500 differentially expressed circRNAs in medulloblastoma (MB) discovery (a) and in MB validation data set (b), hierarchical clustering by average Pearson dissimilarity. Subtypes were defined through methylation data. **c+d**. Boxplots of normalized circRNA expression in combined MB cohorts (discovery and validation data sets together) *versus* healthy brain samples. circEYA1 (c) and circNDST3 (d). Tukey`s HSD adjusted p-values: *=p<0.05; **=p<0.01; ***=p<0.001 (details see Table S1).
