# Supplementary figures and images for: Circular RNA profiling distinguishes medulloblastoma groups and shows aberrant RMST overexpression in WNT medulloblastoma
Source: Acta Neuropathol. 2021 Apr 17;141(6):975–8. doi: 10.1007/s00401-021-02306-2 (PMC8113310; doi:10.1007/s00401-021-02306-2)

**a**

CircFIRRE Combined Cohort  
chrX:130883333-130928494

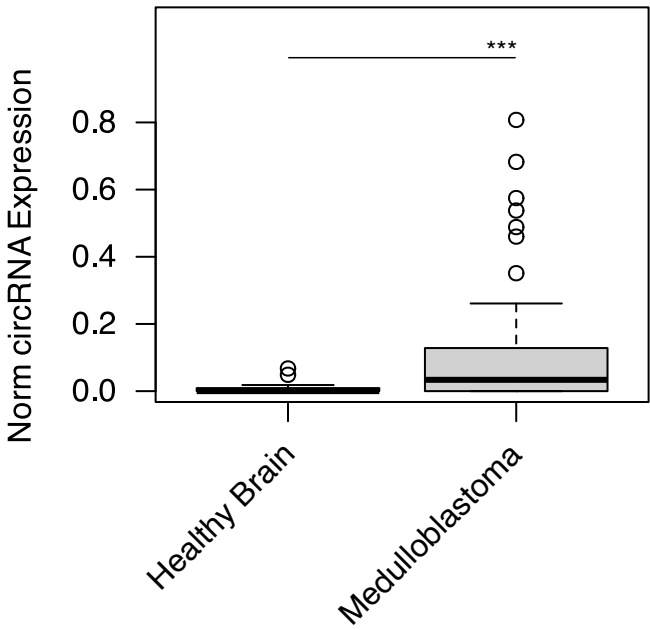

**b**

CircRNF220 Combined Cohort  
chr1:44877652-44878394

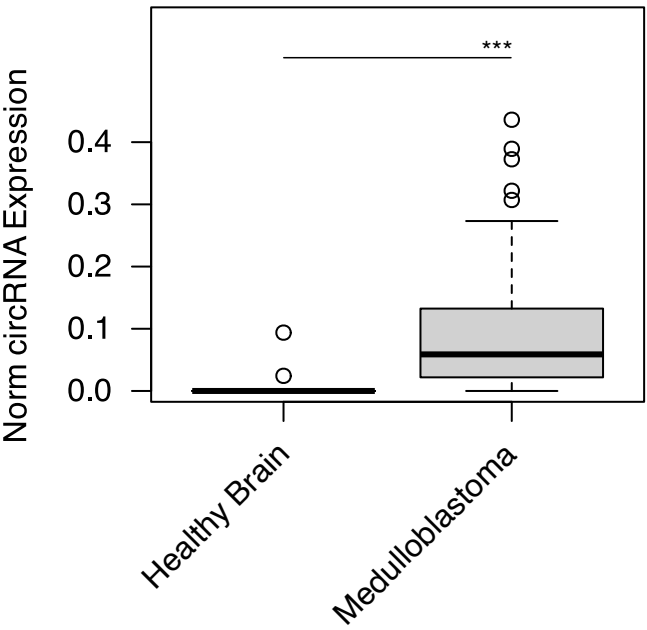

Supplement: Supplementary file 2 — Supplementary file2 (PDF 33 KB) [file 401_2021_2306_MOESM2_ESM.pdf]

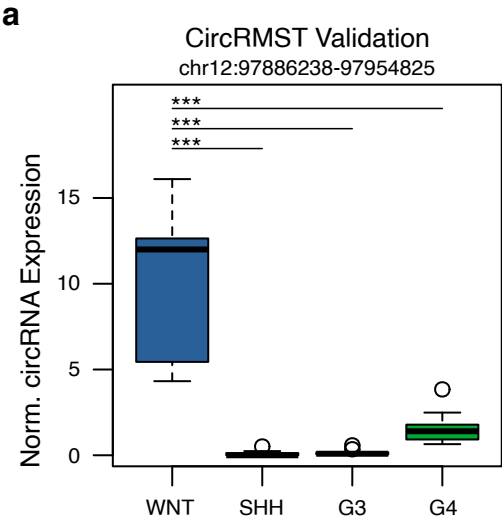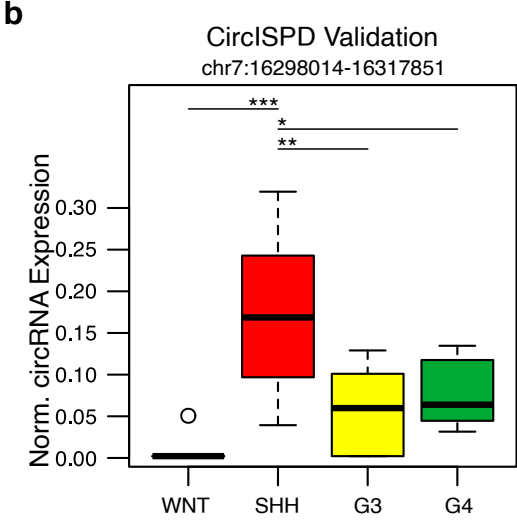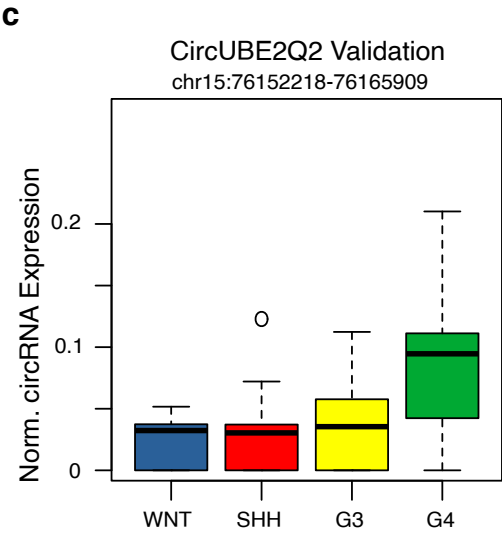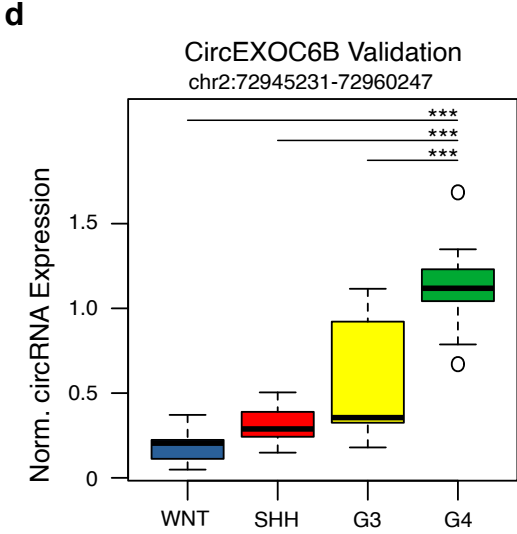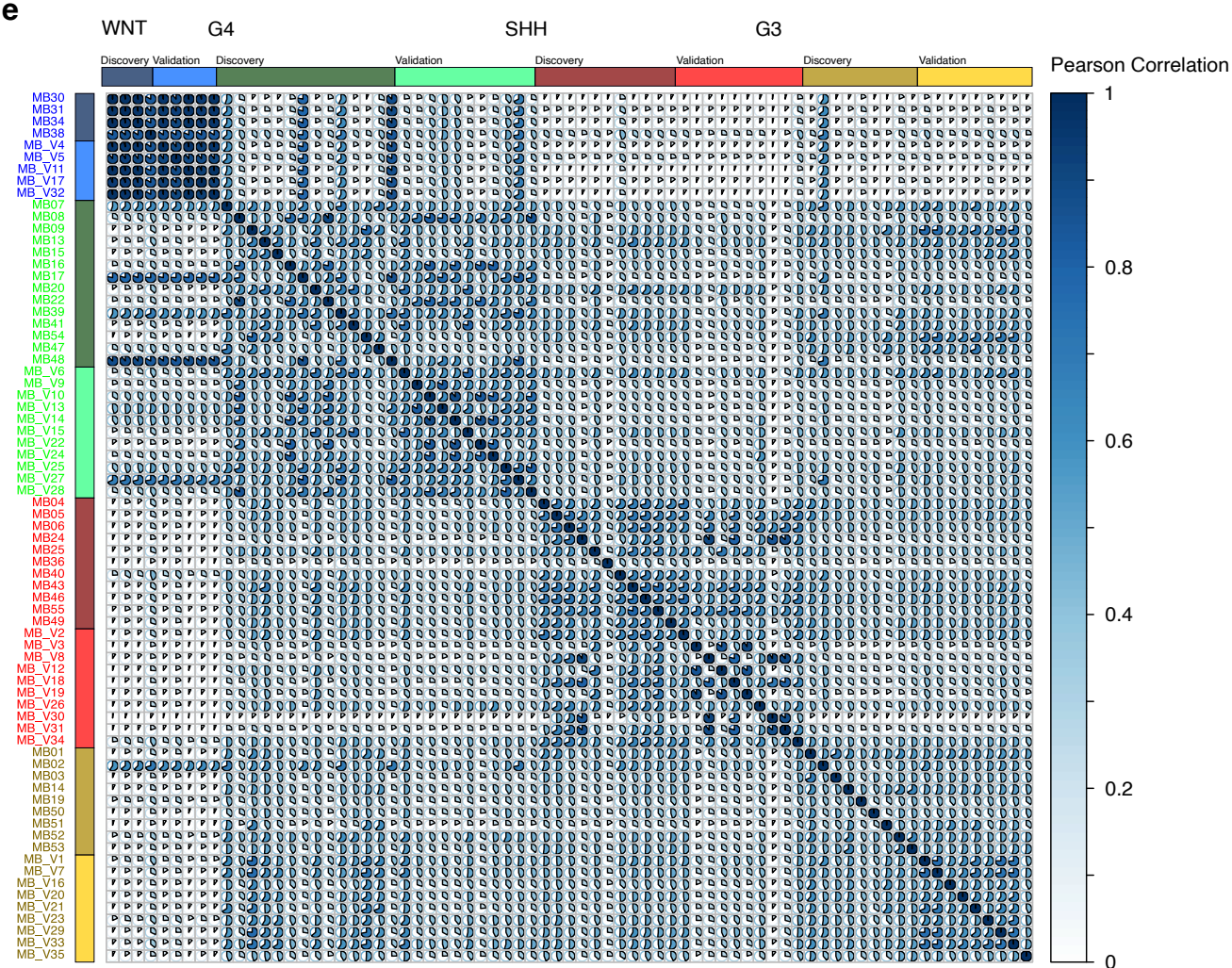

Supplement: Supplementary file 3 — Supplementary file3 (PDF 2997 KB) [file 401_2021_2306_MOESM3_ESM.pdf]

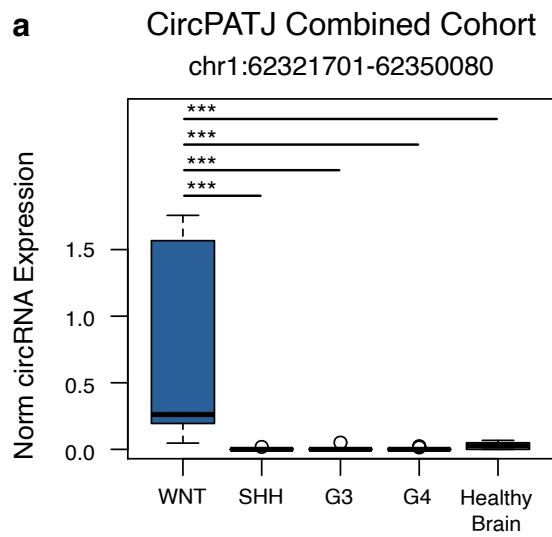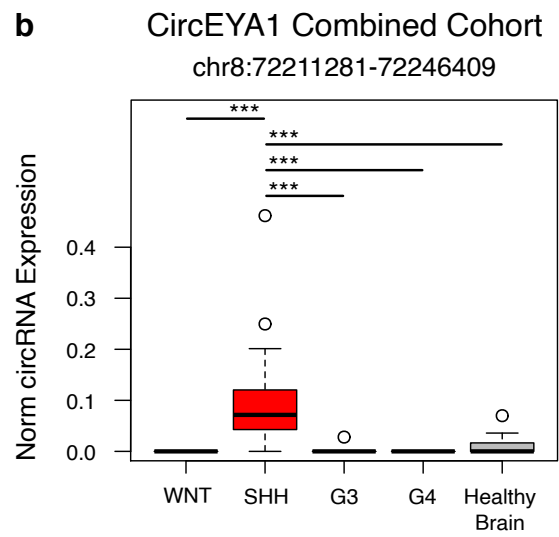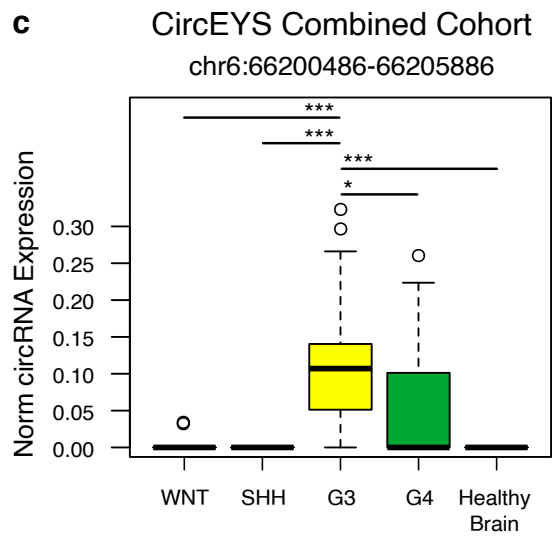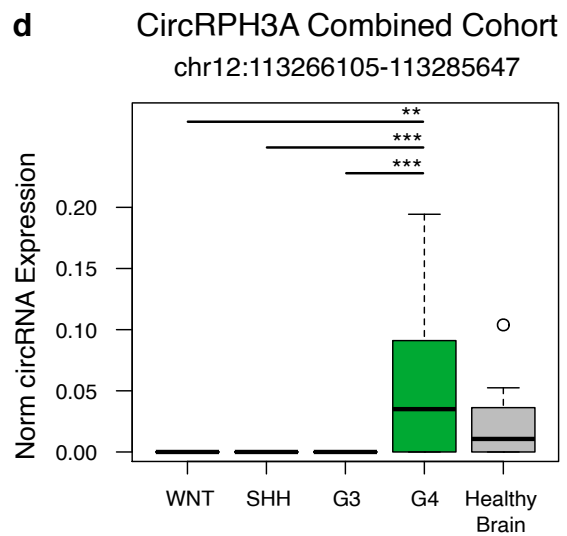

Supplement: Supplementary file 4 — Supplementary file4 (PDF 23 KB) [file 401_2021_2306_MOESM4_ESM.pdf]

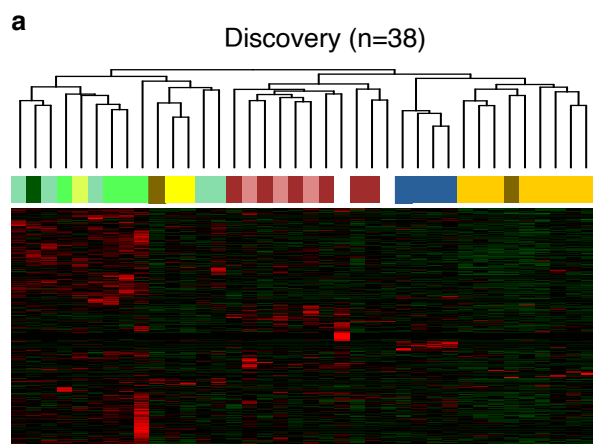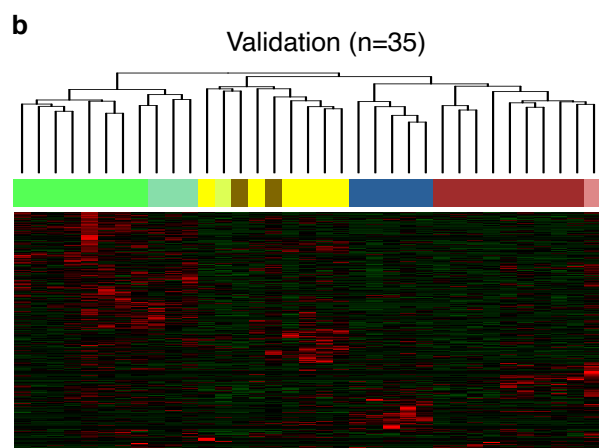

DNA Methylation Subtypes

- WNT
- SHH CHL AD
- SHH INF
- G3 II
- G3 III
- G3 IV
- G3 V
- G4 VI
- G4 VII
- G4 VIII
- NA

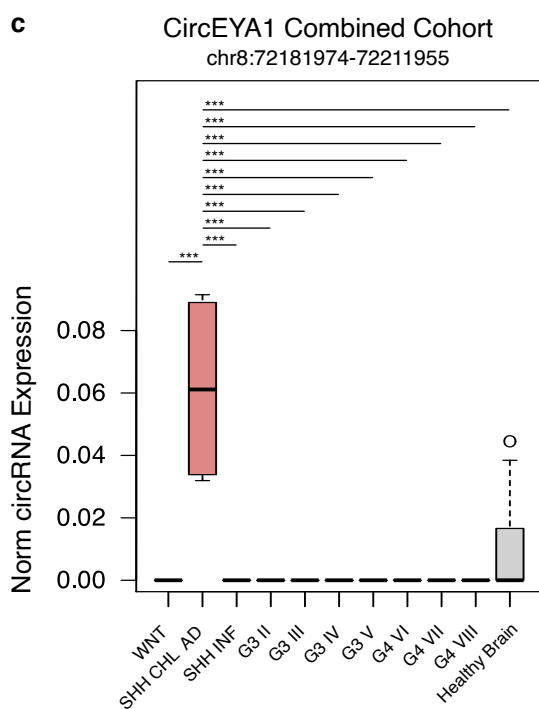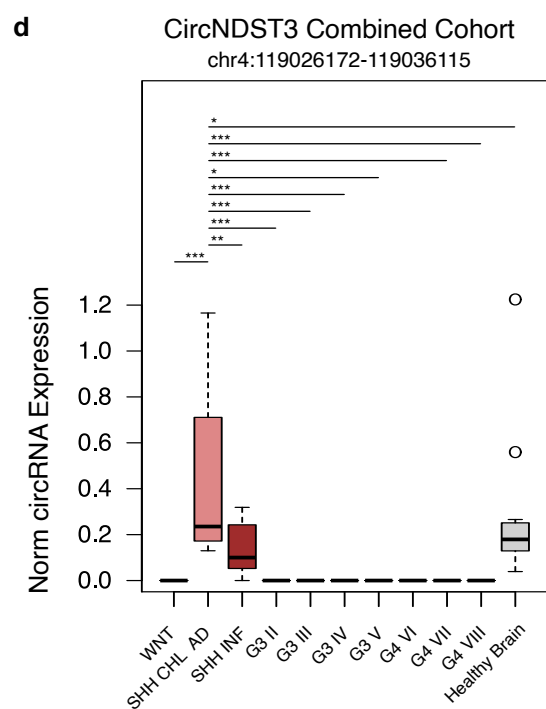

Supplement: Supplementary file 5 — Supplementary file5 (PDF 618 KB) [file 401_2021_2306_MOESM5_ESM.pdf]
